# Romanian wild boars and Mangalitza pigs have a European ancestry and harbour genetic signatures compatible with past population bottlenecks

**DOI:** 10.1038/srep29913

**Published:** 2016-07-15

**Authors:** A. Manunza, M. Amills, A. Noce, B. Cabrera, A. Zidi, S. Eghbalsaied, E. Carrillo de Albornoz, M. Portell, A. Mercadé, A. Sànchez, V. Balteanu

**Affiliations:** 1 Department of Animal Genetics, Center for Research in Agricultural Genomics (CSIC-IRTA-UAB-UB), Campus de la Universitat Autònoma de Barcelona, Bellaterra, 08193 Spain; 2 Transgenesis Center of Excellence, Isfahan (Khorasgan) Branch, Islamic Azad University, Isfahan, Iran; 3 Departament de Ciencia Animal i dels Aliments, Universitat Autònoma de Barcelona, Bellaterra, 08193 Spain; 4 Faculty of Animal Science and Biotechnologies and Institute of Life Sciences, University of Agricultural Sciences and Veterinary Medicine, Cluj-Napoca 400372, Romania

## Abstract

We aimed to analyse the genetic diversity of Romanian wild boars and to compare it with that from other wild boar and pig populations from Europe and Asia. Partial sequencing of the mitochondrial encoded cytochrome b (*MT-CYB*) gene from 36 Romanian wild boars and 36 domestic pigs (Mangalitza, Bazna and Vietnamese breeds) showed that the diversity of Romanian wild boars and Mangalitza pigs is fairly reduced, and that most of the members of these two populations share a common *MT-CYB* haplotype. Besides, in strong contrast with the Bazna animals, Romanian wild boars and Mangalitza swine did not carry Asian variants at the *MT-CYB* locus. The autosomal genotyping of 18 Romanian wild boars with the Illumina Porcine SNP60 BeadChip revealed that their genetic background is fundamentally European, even though signs of a potential Near Eastern ancestry (~25%) were detectable at K = 4 (the most significant number of clusters), but not at higher K-values. Admixture analysis also showed that two wild boars are of a hybrid origin, which could be explained by the mating of feral animals with domestic pigs. Finally, a number of Romanian wild boars displayed long runs of homozygosity, an observation that is consistent with the occurrence of past population bottlenecks and the raise of inbreeding possibly due to overhunting or to the outbreak of infectious diseases.

High throughput genotyping methods are essential for the ascertainment of the demographic and selective forces that have shaped wild boar diversity throughout time. So far, wild boar populations from China^1^, the Iberian Peninsula^2^,^3^, Northwest Europe^4^,^5^ and Near East^3^ have been analysed. Several inferences can be made on the basis of these studies. First, the amount of genetic diversity in European and Near Eastern wild boars^3^,^4^ is generally lower than that of domestic pigs, probably as a consequence of a sustained demographic decline produced by overhunting and habitat loss^6^. This outcome might be also explained by the fact that the SNP discovery panel used to design the Porcine SNP60 BeadChip was mostly based on the variation of domestic pigs rather than wild boars, a circumstance that may lead to the underestimation of the diversity of the latter. Second, wild boar populations are markedly structured (F_ST_ ~0.04–0.2), a feature that may be related with spatial fragmentation produced by human activities and landscape barriers^3^,^5^. Third, wild boars have been introgressed by domestic specimens to some extent^4^, and this might have enhanced their prolificacy and contribute to their rapid demographic expansion.

The genetic characterization of European wild boars has been mostly focused on those living in its Western half. However, the Balkans is a particularly critical area to be explored because this was one of the main wild boar refugia during the Last Glacial Maximum^7^,^8^. The short geographic distance between the Balkans and the Near East reinforces the interest of investigating whether the gene pool of Balkan wild boars is exclusively European or, conversely, it has an Asian influence. Mitochondrial studies have revealed that the maternal ancestry of wild boars from the Balkans is fundamentally European^7^,^8^,^9^,^10^ even though few individuals harbouring Near Eastern haplotypes have been identified^7^,^9^. Unfortunately, such comparisons have not yet been extended to the autosomal genome. One goal of the current work is to characterize the genome-wide and mitochondrial diversity of Romanian wild boars and compare it with that of wild boar and pig populations from Europe and Asia. This approach could provide valuable information about the origins of Romanian wild boars. Another objective was to investigate if the demographic recession that Eastern European wild boar populations experienced during the 17^th^–20^th^ centuries due to climate cooling, habitat destruction by human exploitation and overhunting^9^,^11^,^12^ has left a recognizable signature at the genomic level. With this purpose, we have analysed the size and genomic distribution of runs of homozygosity (ROH) which, as evidenced in pigs^11^, are a powerful source of information of past demographic changes.

## Results and Discussion

### Patterns of diversity in Romanian wild boars and comparative analysis with other wild and domestic pig populations from Europe and Asia

**Figure 1:**
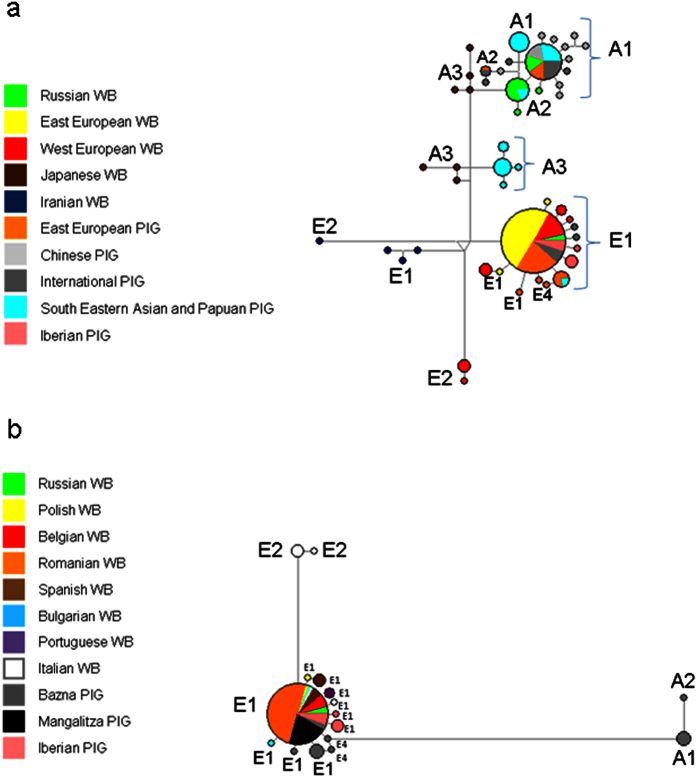
(**a**) Median-joining network depicting the genetic relationships amongst wild boars and pigs sampled in Europe and Asia on the basis of mitochondrially encoded cytochrome b sequences (*MT-CYB*). We have assigned *MT-CYB* haplotypes to eight European (E1, E2, E3 and E4) and Asian (A1, A2, A3 and A4) haplogroups defined in a previous study on the basis of information provided by six diagnostic mutations^14^ The following populations have been included in the network: Wild boars from Russia, East Europe (Romania, Poland and Bulgaria), West Europe (Italy, Spain and Portugal), Japan and Iran; and pigs from East Europe (Bazna and Mangalitza), Spain (Iberian breed), China, and South Eastern Asia (Vietnam and Indonesia) and Papua New Guinea plus two breeds with an international distribution (Large White and Landrace) 1(**b**). Median-joining network including *MT-CYB* sequences from European wild boars and pigs.

The median-joining network of mitochondrially encoded cytochrome b (*MT-CYB*) sequences listed in Tables S1 and S2 showed that all Romanian wild boars and Mangalitza pigs share the same European *MT-CYB* haplotype (Fig. 1). In contrast, *MT-CYB* sequences from Russian wild boars were mainly found in the Far East cluster, which is somewhat not surprising as these animals were sampled in the Primorsky Krai region in the Easternmost fringe of Russia^13^. Bazna pigs were distributed, in similar proportions, within the European and the Far East groups. This finding is not unexpected, since the Bazna breed originated by crossing Mangalitza and Berkshire pigs, and the latter carry Asian alleles at high frequencies^14^. Consistent with this finding, many *MT-CYB* sequences from commercial pig breeds clustered in the Far East group (Fig. 1), as evidenced in many previous studies^15^,^16^.

Mitochondrial variation was fairly small in the set of Romanian pigs and wild boars screened in our analysis (Table 1). Likewise, the Mangalitza pigs employed in our study also showed little mitochondrial variation. This could be explained by the particular origin of this small, old and closed population of only 60 animals which is currently being managed in the framework of a conservation programme. Notably, studies involving a much larger number of Mangalitza pigs have also evidenced that the genetic variability whitin this breed is fairly limited, and certainly much lower than that of commercial breeds as for example Duroc and Piétrain^17^,^18^. At the autosomal level, the observed (H_o_) and expected (H_e_) heterozygosities of pig and wild boar populations were not substantially different, although Near Eastern wild boars showed a slightly reduced diversity if compared to their European counterparts (Table 2). The expected heterozygosities estimated by us in Western (H_e_ = 0.29) and Eastern (H_e_ = 0.31) European wild boars were fairly similar. Other authors have reported H_e_ values ranging between 0.16–0.20^4^ and 0.34–0.41^5^ in Northwest European wild boars. Remarkably, 4 out of the 5 comparisons published to date, described genome-wide H_o_ with larger values than those detected in H_e_. This trend could reflect the interbreeding between individuals from different populations involving wild boar restocking or pig introgression. Either way, it is necessary to highlight that diversity estimates based on the Porcine SNP60 BeadChip are subjected to ascertainment bias *i.e.* this genotyping tool underestimates the variation of populations distantly related with the ones used to design the chip.

**Figure 2:**
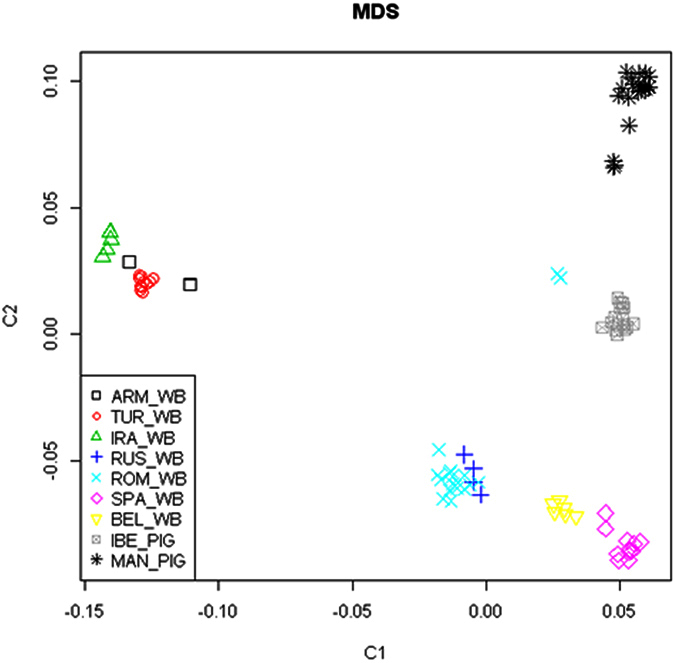
Multidimensional scaling plot based on genome-wide identity-by-state pairwise distances inferred with PLINK. This graph highlights the genetic relationships between Near Eastern wild boars (ARM: Armenia, TUR: Turkey, IRA: Iran), Eastern (Russia and Romania) and Western (Belgium and Spain) European wild boars and Iberian (IBE) and Mangalitza (MAN) pigs.

As expected, our multidimensional scaling (MDS) plot was in agreement with the one reported by Manunza and coworkers^3^, as we essentially used the same dataset with the additional inclusion of 18 Romanian wild boars. The strong genetic divergence identified between Near Eastern and European wild boars (Fig. 2) was evident. Besides, Western and Eastern European wild boars clustered apart, and Mangalitza and Iberian pigs also formed two highly differentiated groups. Interestingly, most Romanian and Russian wild boars (sampled near Moscow) grouped very tightly together, thereby evidencing their common ancestry, with the only exception of two Romanian individuals that were located close to Iberian pigs and that, as we will discuss later, are probably hybrids.

### The ancestry of Romanian wild boars and Mangalitza pigs is fundamentally European

The admixture analysis at K = 2 allowed discriminating between the European and the Near Eastern backgrounds, while at K = 3 the European cluster further split down domestic (Iberian and Mangalitza) and wild pigs (Fig. S1). Remarkably, the Iberian pigs had a mixed porcine/wild boar ancestry. Such feature was inferred in former mitochondrial^19^ and autosomal^2^ analyses. It has been suggested that some level of crossbreeding between Iberian pigs and wild boars may have taken place until Medieval times^19^. These hybridization events could have even occurred at more recent times, as during their production cycle, Iberian pigs are allowed to roam freely in the *dehesa* pastures where they graze on gramineous plants, wild legumes and acorns, thus providing a window of opportunity for unintentional crossbreeding with wild boars.

**Figure 3:**
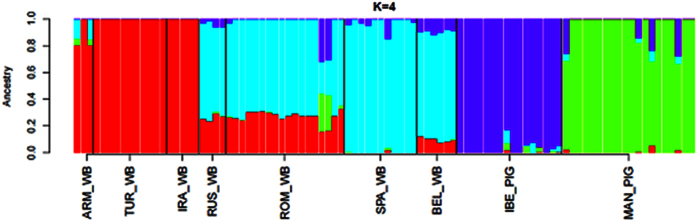
Admixture analysis (K = 4) of wild boars from the Near East (Turkey, Iran and Armenia), East Europe (Russia and Romania), West Europe (Belgium and Spain) and Mangalitza and Iberian pigs. The cross-validation error technique showed that the most significant number of clusters was 4. The complete analysis (K = 2-9) can be found in Fig. S1.

At the most significant K-value (K = 4, Fig. 3), the genetic backgrounds of Near Eastern and European wild boars and Mangalitza and Iberian pigs were clearly distinguishable. Interestingly, Russian and Romanian wild boars showed traces (~25%) of a putative Near Eastern background also present, although to a lower extent, in Belgian wild boars (Fig. 3). This result, however, was not replicated at higher K-values (K ≥ 6, Fig. S1). In consequence, we must conclude that the gene pool of Romanian wild boars is fundamentally European even though a potential, yet less significant, Near Eastern ancestry cannot be ruled out. Indeed, Kusza and coworkers^9^ sampled 254 wild boars from Eastern Europe and identified one Russian specimen that carried a mitochondrial Near Eastern haplotype. Similarly, Near Eastern haplotypes were identified in wild boars from the island of Samos^7^, which is located close to Turkey. The presence of Near Eastern mitochondrial haplotypes in Russian wild boars might be due to past human-mediated translocations *e.g.* wild boars from the Caucasus were released in Novgorod in 1971 to provide huntable game^9^. As wild boars can also migrate to distant locations^20^, a scenario of natural migration can be also envisaged. The main evidence that this gene flow might not be very ancient is provided by the fact that European pigs do not carry Near Eastern genetic signatures^15^. In line with this evidence, our mitochondrial analysis does not reflect the clustering of Bazna or Mangalitza pigs in the Near Eastern clade, and extensive mitochondrial surveys have not uncovered the existence of such relationships in modern pigs^15^,^16^. Although ancient DNA analyses have demonstrated the entry of Near Eastern domestic pigs into Romania during the Neolithic^21^, such event did not leave a long-term footprint in the gene pools of native Romanian porcine breeds.

Noteworthy, the admixture analysis evidenced the existence of two hybrid individuals, from the Covasna county, with a mixed porcine and wild boar ancestry that grouped close to the Iberian pig in the MDS plot (Fig. 2). Nevertheless, the exact source of this porcine introgression is currently unknown. Goedbloed and coworkers^4^ investigated the presence of domestic alleles in 88 Northwest European wild boars and, despite the fact that pig farming practices in this geographic location are intensive and indoors, they found proof of admixture in 10% of the analysed specimens. Hybridization events between pigs and wild boars might not be uncommon in Romania. For instance, a four-year research (2005–2009) in the commune of Bârzava (Arad County, Romania) revealed that as many as 25% pigs were hybrids^22^. The authors link their findings to the traditional pastoral pig management in certain areas of Romania (mainly the Danube Delta and Valley) where wild boars are abundant^22^. Indeed, swine are allowed to roam free throughout the year in pastures near the villages (except at night, when they are kept in sheds). Thus, sexually receptive sows may attract wild boars living in the neighbouring forests and unintentional mating may occur^22^. On the other hand, the intentional release of hybrid individuals to restock areas where wild boar populations have been depleted has been reported in Romania^22^. Crossbreeding between hybrid boars and wild boar females could have important consequences on wild boar demography, as it contributes to the expansion of the wild populations (domestic pigs are more prolific than wild boars) and might thus become an agricultural pest with a clear negative impact on the survival of ground-nesting birds and small mammals. Besides, the uncontrolled mating of wild and domestic pigs could contribute to the spread of certain infectious diseases, as swine fever, pseudorabies and brucellosis, and to a loss of local adaptation of wild boars (outbreeding depression).

### Assessing the demography of Romanian wild boars and other European and Asian wild and domestic pig populations through the analysis of runs of homozygosity

**Figure 4:**
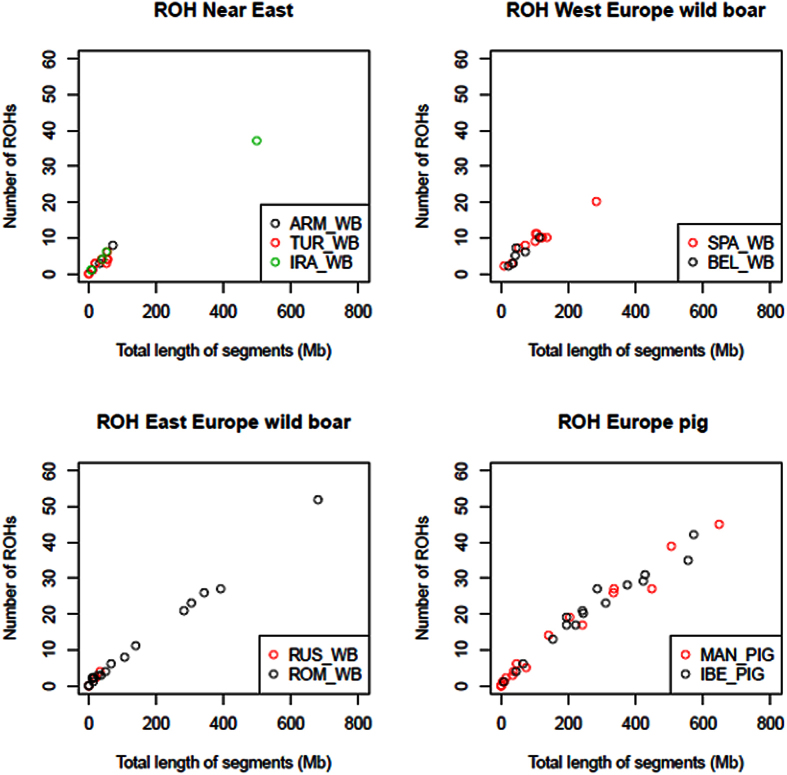
Runs of homozygosity (ROH) complement (total length and number) in the individual genomes of Near Eastern, East European and West European wild boars and Iberian and Mangalitza pigs.

**Figure 5:**
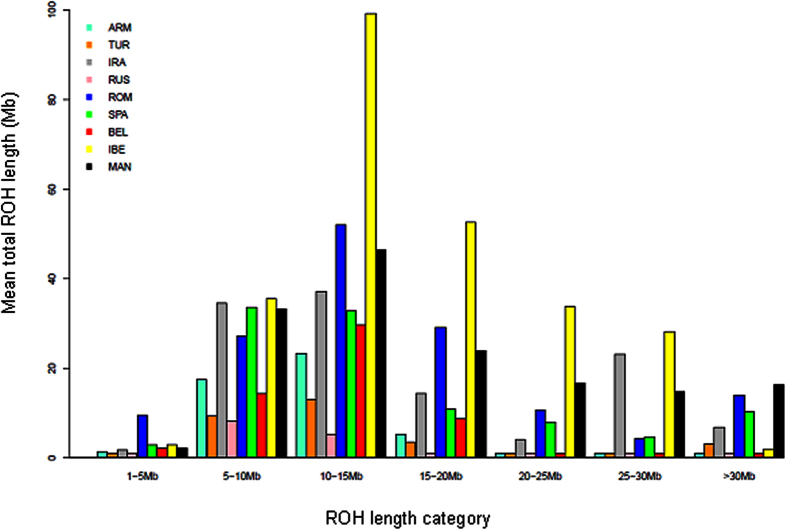
Regional distribution of ROH. The Y-axis indicates the average number of megabases covered by each ROH class (classified according to their size) in Near Eastern, East European and West European wild boars and Iberian and Mangalitza pigs.

As shown in Figs 4 and 5, we have characterized the length and distribution of ROH in Romanian wild boars as well as in other domestic and wild pig populations typed with the Illumina Infinium HD Porcine SNP60 BeadChip^3^. We have observed that a significant number of Romanian wild boars and Mangalitza and Iberian pigs have ROH with a length that ranges between 300 and 800 Mb. On the contrary,such long ROH are exceptional in Near Eastern and Western European wild boars (Fig. 4). Consistently, in the Mangalitza and Iberian pigs, and to a lesser extent in Romanian wild boars, the 15–30 Mb ROH class has a larger genome coverage than in other populations (Fig. 5). It should be noted that the Illumina Infinium HD Porcine SNP60 BeadChip cannot detect efficiently small sized ROH^11^, so our estimates of ROH total length may be biased downwards. On the contrary, large ROH may be overrepresented in our dataset because, as previously said, the small ones are missed. Indeed, in a previous study^23^ it was found that the average ROH length in Balkan wild boars was ~5 Mb, while our data show that the ROH category with the largest genome coverage corresponds to that with a size between 10–15 Mb (Fig. 5)

The genomic distribution and length of ROH recapitulates faithfully the demography of wild and domestic pig populations^11^. European wild boars underwent a strong founder effect as a consequence of their initial dispersal from South East Asia 1.2 Mya^11^. Subsequently, another population drop took place 50 kya and continued thereafter^11^. Marked population size reductions have been documented in several European countries. For instance, in Italy, at the beginning of the 20^th^ century, wild boar distribution was restricted to Sardinia and certain Central-Southern parts of the country^24^, while in England they became extinct in the 13^th^ century^25^. In Romania, there are currently around 62,000 wild boars, but during the 1955–75 period this population was four times smaller^26^, probably because excessive hunting during and after World War II. Another sharp population decrease during the mid eighties has been reported^26^, possibly as a consequence of a classical swine fever outbreak. This may explain the limited variability found at the mitochondrial level and the presence of individuals that may be highly inbred, as suggested by ROH data (Fig. 4).

Our results show that total ROH length and sizes are relatively small in Near Eastern and Western European wild boars (Figs 4 and 5), which is consistent with reduced inbreeding in recent times. Natural migratory events as well as intentional wild boar translocation and restocking of forestlands where this species became extinct^27^,^28^ may have counteracted the effects of factors that tend to reduce population size. Indeed, wild boars can travel considerable distances (sometimes >250 km), particularly when males reach sexual maturity or before the beginning of the mating season^20^, and multiple paternities in a single litter may occur^29^,^30^. The occasional admixture between pigs and wild boars, could have also augmented the diversity of the latter, since the main porcine commercial breeds carry Asian alleles to a significant extent^14^. Finally, the decreased use of agricultural and forested lands by humans may have also favoured the growth of wild boar populations.

Mangalitza pigs displayed the largest ROHs sizes and total length (Figs 4 and 5). Such findings are consistent with the endangered status of this breed in Romania, which, in the last fifty years, has been facing extinction. As previously said, the Mangalitza pigs employed in our study correspond to a closed population managed at a single farm at the Cluj county, so inbreeding levels may be rather high. This could have detrimental consequences on reproductive performance and viability^31^. Similarly, a number of the Iberian pigs analysed in the current work showed a high frequency of long ROH. A previous study showed that in Iberian pigs the fraction of the genome covered by ROH is modest, although there were individuals with a high ROH genome coverage^2^. Such heterogeneity, also evident in our dataset (Fig. 4), may be attributable to the existence of many Iberian lines and strains with distinct demographic histories.

## Conclusions

Our analysis of the autosomal diversity of Romanian wild boars has demonstrated that their autosomal genetic background is essentially European, although a potential, and much less significant, influence of the Near Eastern gene pool cannot be ruled out. A whole genome sequencing approach would be definitely needed to assess if Near Eastern alleles have differential frequencies in Western and Eastern European wild boars. We have also identified two individuals (*i.e.* 10% of the total sample) with enough evidence of porcine blood introgression. Similar findings have been obtained in Northwest European wild boars^4^, which makes clear the existence of a widespread gene flow between domestic and wild pigs. This could have favoured the expansion of wild boar populations, an outcome that may cause the destruction of crops and threat the preservation of other species preyed by wild boars. Finally, the limited mitochondrial variation of Romanian wild boars and the presence of several individuals with a substantial part of their genomes covered with long ROH are suggestive of the occurrence of past population bottlenecks and recent inbreeding.

## Materials and Methods

### Ethics statement

Hair extraction from domestic pigs was performed in accordance with the rules of the Research Bioethics Commission of the University of Agricultural Sciences and Veterinary Medicine at Cluj-Napoca (Romania), and all protocols were approved by this institution. Regarding wild boars, all samples were retrieved from individuals previously killed as a consequence of hunting activities completely unrelated with our project, so a permission of the Ethics Committeee referred above did not apply.

### Wild boar and domestic pig sampling and isolation of nucleic acids

Romanian wild boar samples (N = 36) were obtained at 12 representative locations listed at Table S1. Tissue samples (muscle and kidney) were collected during the legal hunting season and submerged in RNAlater preserving solution (Life Technologies, Barcelona, Spain). Genomic DNA was isolated with a phenol chloroform protocol. In this way, 30 mg tissue was submerged in 500 μl lysis buffer (50 mM Tris Base pH 8, 20 mM EDTA, 2% SDS) and 15 μl of proteinase K (10 mg/ml) and incubated overnight at 56 °C. Subsequently, we added an equal volume (1:1) of a phenol:chloroform:isoamyl alcohol (25:24:1) mixture, inverted the tube several times and centrifuged at 13,000 rpm for 15 min. The upper layer was transferred to a fresh tube. Subsequently, DNA was precipitated by adding 2 volumes of ice-cold ethanol and 0.1 volumes 2 M NaCl and centrifuging at 13,000 rpm for 30 min. The supernatant was discarded, and an additional step, aimed to remove salt contamination, was carried out by adding 70% ethanol and centrifuging at 13,000 rpm for 10 min. Finally, the DNA pellet was dissolved in 50 μl Milli-Q water and kept at −20 °C.

Total DNA was also extracted from hair shafts corresponding to Mangalitza (N = 12), Vietnamese (N = 10) and Bazna (N = 14) pigs sampled at Romania (Table S1). We used the DNeasy Blood & Tissue Kit (Qiagen, Barcelona, Spain) in accordance with the instructions of the manufacturer. We generated this dataset with the purpose of comparing the diversity of Romanian wild boars, the main focus of our study, with that of local pig breeds (Bazna and Mangalitza) plus a few representatives of the Far Eastern gene pool (Vietnamese pigs).

### Sequencing of a fragment of the mitochondrially encoded cytochrome b gene

A fragment of the *MT-CYB* gene was amplified with a set of previously reported primers^32^. Polymerase chain reactions (PCRs) were performed in a 25 μl volume including 2.5 μl of 10
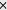
PCR buffer, 1.5 mM MgCl_2_, 0.5 μM of each primer^32^, 0.2 mM of each dNTP, 50 ng genomic DNA and 1.25 U BioTaq DNA polymerase (*Bioline, London, United Kingdom*). Thermocycling included a denaturation step at 94 °C for 5 min, followed by 35 cycles of denaturation at 94 °C for 1 min, annealing at 59 °C for 1 min and extension at 72 °C for 2 min. Finally, an extension step at 72 °C for 10 min was carried out. Amplicons were purified with the ExoSAP-IT PCR Cleanup kit (Affymetrix, Santa Clara, CA) and sequenced in both directions with the same primers used in the amplification step. Sequencing reactions were prepared with the Big Dye Terminator Cycle Sequencing Kit v1.1 (Applied Biosystems, Foster City, CA) and electrophoresed in an ABI 3730 DNA Analyzer (Applied Biosystems, Foster City, CA). Chromatograms were visually inspected and edited with the SeqScape Software v3.0, (Life Technologies, Barcelona, Spain).

### High throughput genotyping with the Illumina Infinium HD Porcine SNP60 BeadChip

A total of 18 Romanian wild boar DNA samples were genotyped with the Illumina Infinium HD Porcine SNP60 BeadChip (Illumina, San Diego, CA), following the protocols reported in a previous publication^3^. Quality genotyping analyses were performed with the GenomeStudio software (Illumina). The GenCall score cutoff was set to 0.15 and the average call rate was 99%. Data filtering was done with PLINK v. 1.07^33^ and markers showing departures from Hardy-Weinberg expectations (*P*-value < 0.001), a minor allele frequency below 0.05 or a rate of missing genotypes >10% were removed. Markers mapping to the X chromosome or in linkage disequilibrium (they were detected with the PLINK –*indep 50 5 2* command) were also eliminated. After these filtering steps, the final dataset included a total of 10,739 SNPs.

### Population genetics analyses

A total of 72 *MT-CYB* sequences obtained in the current study (Table S1) plus 112 retrieved from GenBank (Table S2) were employed in mitochondrial analyses. Sequences were aligned to a reference *MT-CYB* sequence (Genbank AJ002189^34^) and trimmed to cover an 895 bp region. With this dataset, we built a median-joining network with the Network 4.6 software^35^. Nucleotide and haplotype diversities and the number of haplotypes were calculated with the DnaSP v5 software^36^.

The analysis of autosomal variation comprised 18 Romanian wild boars plus a number of wild boar and pig specimens described in a previous work^3^. More specifically, we used 60 K SNP data from Near Eastern (N = 19), Russian (N = 4) and West European (Belgium and Spain, N = 17) wild boars as well as from Iberian (N = 16) and Mangalitza pigs (N = 20) to calculate observed and expected heterozygosities. Moreover, we built, with PLINK v. 1.07^33^, an MDS plot based on a genome-wide identity-by-state pairwise distances matrix, whereas population structure was inferred with the Admixture v. 1.23 package^37^. This software is based on a statistical model, very similar to that of Structure, that models the probability of the observed genotypes by taking into account ancestry proportions as well as population allele frequencies^37^. Though Structure and Admixture rely on the same maximum likelihood model, Structure takes a Bayesian approach and uses a Markov Chain Monte Carlo algorithm to sample the posterior distributions of the parameters to be estimated^31^. In contrast, Admixture computes maximum likelihood estimates of the parameters and, in consequence, it is much faster and it can accomodate many more markers^37^. Admixture identifies the optimal number of clusters (K-value) as that with the lowest cross-validation error^38^. For the termination criteria we used default parameters.

The analysis of ROH was carried out with PLINK v. 1.07^33^. This software uses a sliding window to identify long contiguous homozygous segments across the genome. In order to avoid the detection of spurious stretches generated by chance, we filtered markers that were in strong linkage disequilibrium, as suggested by Purcell and coworkers^33^. The approach implemented in PLINK to detect ROH is based on a sliding window that identifies homozygous segments by assessing the genotypic status of each consecutive SNP. In this way, PLINK estimates the proportion of completely homozygous windows that contain a given SNP, a parameter defined by the homozyg-window-threshold command that, in our case, was set to 0.001 (if 0.1% of the windows were homozygous then the SNP was included in a ROH). In addition, ROHs were called when they had a minimum size of 50 SNPs (-homozyg-snp 50) and 1000 kb (–homozyg- kb 1000), and the minimum density of SNPs was of 1 SNP every 5000 kb (–homozyg-density 5000). We allowed 1 heterozygous SNP (–homozyg-window-het 1) and 5 missing SNPs (–homozyg-window-missing 50) per ROH because this approach increases the power of detecting truly autozygous segments.

## Additional Information

**How to cite this article**: Manunza, A. *et al*. Romanian wild boars and Mangalitza pigs have a European ancestry and harbour genetic signatures compatible with past population bottlenecks. *Sci. Rep.* **6**, 29913; doi: 10.1038/srep29913 (2016).

## Supplementary Material

Supplementary Information

## Figures and Tables

**Table 1:** Levels of mitochondrial diversity in several wild boar (WB) and pig populations.

Population	Number of sequences	Nucleotide diversity	Haplotype diversity	Haplotype number
Iranian WB	4	0.0069	1.000	4
**Romanian WB**	31	0.0000	0.000	1
West European WB	15	0.0056	0.838	6
Russian WB	17	0.0050	0.728	5
**Mangalitza pig**	12	0.0000	0.000	1
**Bazna pig**	14	0.0081	0.857	7
Iberian pig	8	0.0014	0.857	5
Landrace pig	10	0.0082	0.800	5
Large White pig	7	0.0069	0.952	6
Japanese WB	7	0.0051	1.000	7
Indonesian pig	17	0.0053	0.801	6
Chinese pig	13	0.0022	0.923	10
**Vietnamese pig**	10	0.0039	0.511	3

Sequences generated in the current work are marked in bold. Southern European WB were sampled in Italy, Spain and Portugal.

**Table 2:** Observed (H_o_) and expected (H_e_) heterozygosities in several wild boar and pig populations.

Population	H_o_	H_e_
Iranian WB	0.284	0.249
Romanian and Russian WB (East Europe)	0.325	0.313
Belgian and Spanish WB (West Europe)	0.301	0.296
Iberian pig	0.285	0.318
Mangalitza pig	0.351	0.304
