# Supplementary material for: Romanian wild boars and Mangalitza pigs have a European ancestry and harbour genetic signatures compatible with past population bottlenecks
Source: Sci Rep. 2016 Jul 15;6:29913. doi: 10.1038/srep29913 (PMC4945946; doi:10.1038/srep29913)

**Romanian wild boars and Mangalitza pigs have a European ancestry and harbour genetic signatures compatible with past population bottlenecks**

A. Manunza ¶, M. Amills ¶*, A. Noce ¶, B. Cabrera ¶, A. Zidi ¶, S. Eghbalsaied §, E. Carrillo de Albornoz ¶, M. Portell ¶, A. Mercadé †, A. Sànchez ¶, V. Balteanu ¥

¶ Department of Animal Genetics, Center for Research in Agricultural Genomics (CSIC-IRTA-UAB-UB), Campus de la Universitat Autònoma de Barcelona, Bellaterra, 08193 Spain; § Young Researchers and Elite Club, Isfahan (Khorasgan) branch, Islamic Azad University, Isfahan, Iran; † Departament de Ciencia Animal i dels Aliments, Universitat Autònoma de Barcelona, Bellaterra, 08193 Spain; ¥ Institute of Life Sciences, University of Agricultural Sciences and Veterinary Medicine, Cluj-Napoca 400372, Romania.

Supplementary Table S1. List of wild boar and pigs samples sequenced in the current study

| **Laboratory_ID** | **Status** | **Location** | **GenBank accession code** |
| --- | --- | --- | --- |
| 1R | Wild boar | Romania (Iasi) | KR604750 |
| 9R | Wild boar | Romania (Iasi) | KR604751 |
| 11R | Wild boar | Romania (Constanta) | KR604752 |
| 12R | Wild boar | Romania (Constanta) | KR604753 |
| 17R | Wild boar | Romania (Covasna) | KR604754 |
| 21R | Wild boar | Romania (Covasna) | KR604755 |
| 25R | Wild boar | Romania (Covasna) | KR604756 |
| 27R | Wild boar | Romania (Harghita) | KR604757 |
| 28R | Wild boar | Romania (Harghita) | KR604758 |
| 94R | Wild boar | Romania (Alba) | KR604759 |
| 30R | Wild boar | Romania (Harghita) | KR604760 |
| 31R | Wild boar | Romania (Harghita) | KR604761 |
| 33R | Wild boar | Romania (Harghita) | KR604762 |
| 35R | Wild boar | Romania (Harghita) | KR604763 |
| 45R | Wild boar | Romania (Harghita) | KR604764 |
| 48R | Wild boar | Romania (Harghita) | KR604765 |
| 49R | Wild boar | Romania (Harghita) | KR604766 |
| 62R | Wild boar | Romania (Bistrita) | KR604767 |
| 63R | Wild boar | Romania (Bistrita) | KR604768 |
| 65R | Wild boar | Romania (Cluj) | KR604769 |
| 69R | Wild boar | Romania (Cluj) | KR604770 |
| 74R | Wild boar | Romania (Cluj) | KR604771 |
| 79R | Wild boar | Romania (Cluj) | KR604772 |
| 80R | Wild boar | Romania (Cluj) | KR604773 |
| 81R | Wild boar | Romania (Mures) | KR604774 |
| 82R | Wild boar | Romania (Sibiu) | KR604775 |
| 90R | Wild boar | Romania (Cluj) | KR604776 |
| 93R | Wild boar | Romania (Alba) | KR604777 |
| 100R | Wild boar | Romania (Arad) | KR604778 |
| 101R | Wild boar | Romania (Arad) | KR604779 |
| 103R | Wild boar | Romania (Arad) | KR604780 |
| 110R | Wild boar | Romania (Mehedinti) | KR604781 |
| 111R | Wild boar | Romania (Mehedinti) | KR604782 |
| 112R | Wild boar | Romania (Mehedinti) | KR604783 |
| 119R | Wild boar | Romania (Bihor) | KR604784 |
| 127R | Wild boar | Romania (Bihor) | KR604785 |
| B1 | Domestic | Bazna (Cluj) | KR705510 |
| B2 | Domestic | Bazna (Cluj) | KR705511 |
| B3 | Domestic | Bazna (Cluj) | KR705512 |
| B4 | Domestic | Bazna (Cluj) | KR705513 |
| B5 | Domestic | Bazna (Cluj) | KR705514 |
| B6 | Domestic | Bazna (Cluj) | KR705515 |
| B7 | Domestic | Bazna (Cluj) | KR705516 |
| B8 | Domestic | Bazna (Cluj) | KR705517 |
| B9 | Domestic | Bazna (Cluj) | KR705518 |
| B10 | Domestic | Bazna (Cluj) | KR705519 |
| B11 | Domestic | Bazna (Cluj) | KR705520 |
| B12 | Domestic | Bazna (Cluj) | KR705521 |
| B13 | Domestic | Bazna (Cluj) | KR705522 |
| B14 | Domestic | Bazna (Cluj) | KR705523 |
| MRN1 | Domestic | Mangalitza (Cluj) | KR705541 |
| MRN2 | Domestic | Mangalitza (Cluj) | KR705542 |
| MRN3 | Domestic | Mangalitza (Cluj) | KR705543 |
| MRN4 | Domestic | Mangalitza (Cluj) | KR705544 |
| MRN5 | Domestic | Mangalitza (Cluj) | KR705545 |
| MRN6 | Domestic | Mangalitza (Cluj) | KR705546 |
| MRN7 | Domestic | Mangalitza (Cluj) | KR705547 |
| MRN8 | Domestic | Mangalitza (Cluj) | KR705548 |
| MRN9 | Domestic | Mangalitza (Cluj) | KR705549 |
| MRN10 | Domestic | Mangalitza (Cluj) | KR705550 |
| MRN11 | Domestic | Mangalitza (Cluj) | KR705551 |
| MRN12 | Domestic | Mangalitza (Cluj) | KR705552 |
| V1 | Domestic | Vietnamese (Cluj) | KR705556 |
| V4 | Domestic | Vietnamese (Cluj) | KR705557 |
| V5 | Domestic | Vietnamese (Cluj) | KR705558 |
| V6 | Domestic | Vietnamese (Cluj) | KR705559 |
| V7 | Domestic | Vietnamese (Cluj) | KR705560 |
| V8 | Domestic | Vietnamese (Cluj) | KR705561 |
| V12 | Domestic | Vietnamese (Cluj) | KR705562 |
| V13 | Domestic | Vietnamese (Cluj) | KR705563 |
| V14 | Domestic | Vietnamese (Cluj) | KR705564 |
| V15 | Domestic | Vietnamese (Cluj) | KR705565 |

Supplementary Table S2. List of pig and wild boar *MT-CYB* sequences retrieved from GenBank

| **Population** | **GenBank Accession code** | **Status** | **Reference** |
| --- | --- | --- | --- |
| Bulgary | AM492592 | Wild boar | Mona. et al . 2007. Mol. Phylogenet. Evol. 45,757-762 |
| Bulgary | AM492593 | Wild boar | Mona. et al . 2007. Mol. Phylogenet. Evol. 45,757 |
| China, Zizhong | AM492597 | Domestic | Mona. et al . 2007. Mol. Phylogenet. Evol. 45,757 |
| China, Zizhong | AM492598 | Domestic | Mona. et al . 2007. Mol. Phylogenet. Evol. 45,757 |
| China, Zizhong | AM492599 | Domestic | Mona. et al . 2007. Mol. Phylogenet. Evol. 45,757 |
| China, Ziyang | AM492600 | Domestic | Mona. et al . 2007. Mol. Phylogenet. Evol. 45,757 |
| China, Jingtang | AM492601 | Domestic | Mona. et al . 2007. Mol. Phylogenet. Evol. 45,757 |
| China, Jingtang | AM492602 | Domestic | Mona. et al . 2007. Mol. Phylogenet. Evol. 45,757 |
| China, Wenjiang | AM492603 | Domestic | Mona. et al . 2007. Mol. Phylogenet. Evol. 45,757 |
| China, Shangliu | AM492604 | Domestic | Mona. et al . 2007. Mol. Phylogenet. Evol. 45,757 |
| China, Shangliu | AM492605 | Domestic | Mona. et al . 2007. Mol. Phylogenet. Evol. 45,757 |
| China, Huayang | AM492606 | Domestic | Mona. et al . 2007. Mol. Phylogenet. Evol. 45,757 |
| China, Wuhou District, | AM492607 | Domestic | Mona. et al . 2007. Mol. Phylogenet. Evol. 45,757 |
| China, Jingtang | AM492608 | Domestic | Mona. et al . 2007. Mol. Phylogenet. Evol. 45,757 |
| China, Xindu | AM492609 | Domestic | Mona. et al . 2007. Mol. Phylogenet. Evol. 45,757 |
| Iberian | EF061505 | Domestic | Ojeda et al. 2006. Genetics 174,2119-2127 |
| Iberian | EF061503 | Domestic | Ojeda et al. 2006. Genetics 174,2119-2127 |
| Indonesia, Asmat | AM492551 | Feral | Mona. et al . 2007. Mol. Phylogenet. Evol. 45,757 |
| Indonesia, Asmat | AM492552 | Feral | Mona. et al . 2007. Mol. Phylogenet. Evol. 45,757 |
| Indonesia, Bali | AM492561 | Domestic | Mona. et al . 2007. Mol. Phylogenet. Evol. 45,757 |
| Indonesia, Bali | AM492562 | Domestic | Mona. et al . 2007. Mol. Phylogenet. Evol. 45,757 |
| Indonesia, Bali | AM492563 | Domestic | Mona. et al . 2007. Mol. Phylogenet. Evol. 45,757 |
| Indonesia, Bali | AM492564 | Domestic | Mona. et al . 2007. Mol. Phylogenet. Evol. 45,757 |
| Indonesia, Bali | AM492565 | Domestic | Mona. et al . 2007. Mol. Phylogenet. Evol. 45,757 |
| Indonesia, Manokwari | AM492571 | Feral | Mona. et al . 2007. Mol. Phylogenet. Evol. 45,757 |
| Indonesia, Manokwari | AM492572 | Feral | Mona. et al . 2007. Mol. Phylogenet. Evol. 45,757 |
| Indonesia, Manokwari | AM492573 | Feral | Mona. et al . 2007. Mol. Phylogenet. Evol. 45,757 |
| Indonesia, Manokwari | AM492574 | Feral | Mona. et al . 2007. Mol. Phylogenet. Evol. 45,757 |
| Indonesia, Nabire | AM492575 | Feral | Mona. et al . 2007. Mol. Phylogenet. Evol. 45,757 |
| Indonesia, Nabire | AM492576 | Feral | Mona. et al . 2007. Mol. Phylogenet. Evol. 45,757 |
| Indonesia, Nabire | AM492577 | Feral | Mona. et al . 2007. Mol. Phylogenet. Evol. 45,757 |
| Indonesia, Solo-java | AM492629 | Domestic | Mona. et al . 2007. Mol. Phylogenet. Evol. 45,757 |
| Indonesia, Solo-java | AM492630 | Domestic | Mona. et al . 2007. Mol. Phylogenet. Evol. 45,757 |
| Indonesia, Bandung - Java | AM492642 | Domestic | Mona. et al . 2007. Mol. Phylogenet. Evol. 45,757 |
| Indonesia, Melinani - Seram | AM492643 | Feral | Mona. et al . 2007. Mol. Phylogenet. Evol. 45,757 |
| Indonesia, Melinani - Seram | AM492644 | Feral | Mona. et al . 2007. Mol. Phylogenet. Evol. 45,757 |
| Iran | EU531827 | Wild boar | Ramírez et al. 2009. Mol Biol Evol. 26,2061-72 |
| Iran | EU531832 | Wild boar | Ramírez et al. 2009. Mol Biol Evol. 26,2061-72 |
| Iran | EU531833 | Wild boar | Ramírez et al. 2009. Mol Biol Evol. 26,2061-72 |
| Iran | EU531834 | Wild boar | Ramírez et al. 2009. Mol Biol Evol. 26,2061-72 |
| Italy | AM492620 | Wild boar | Mona. et al . 2007. Mol. Phylogenet. Evol. 45,757 |
| Italy | AM492621 | Wild boar | Mona. et al . 2007. Mol. Phylogenet. Evol. 45,757 |
| Italy | AM492622 | Wild boar | Mona. et al . 2007. Mol. Phylogenet. Evol. 45,757 |
| Italy | AM492623 | Wild boar | Mona. et al . 2007. Mol. Phylogenet. Evol. 45,757 |
| Italy | AM492624 | Wild boar | Mona. et al . 2007. Mol. Phylogenet. Evol. 45,757 |
| Italy | AM492625 | Wild boar | Mona. et al . 2007. Mol. Phylogenet. Evol. 45,757 |
| Japan | [AB015067](http://www.ebi.ac.uk/cgi-bin/sva/sva.pl?query=AB015067&search=Go&snapshot=) | Wild boar | Watanabe et al.1999. Mol. Ecol. 8,1509-1512. |
| Japan | [AB015068](http://www.ebi.ac.uk/cgi-bin/sva/sva.pl?query=AB015068&search=Go&snapshot=) | Wild boar | Watanabe et al.1999. Mol. Ecol. 8,1509-1512. |
| Japan | [AB015069](http://www.ebi.ac.uk/cgi-bin/sva/sva.pl?query=AB015069&search=Go&snapshot=) | Wild boar | Watanabe et al.1999. Mol. Ecol. 8,1509-1512. |
| Japan | [AB015070](http://www.ebi.ac.uk/cgi-bin/sva/sva.pl?query=AB015070&search=Go&snapshot=) | Wild boar | Watanabe et al.1999. Mol. Ecol. 8,1509-1512. |
| Japan | [AB015071](http://www.ebi.ac.uk/cgi-bin/sva/sva.pl?query=AB015071&search=Go&snapshot=) | Wild boar | Watanabe et al.1999. Mol. Ecol. 8,1509-1512. |
| Japan | [AB015072](http://www.ebi.ac.uk/cgi-bin/sva/sva.pl?query=AB015072&search=Go&snapshot=) | Wild boar | Watanabe et al.1999. Mol. Ecol. 8,1509-1512. |
| Japan | [AB015073](http://www.ebi.ac.uk/cgi-bin/sva/sva.pl?query=AB015073&search=Go&snapshot=) | Wild boar | Watanabe et al.1999. Mol. Ecol. 8,1509-1512. |
| Mangalitza | JN601066 | Domestic | Cannon et al. 2015. Anim Biotechnol. 26,17-28 |
| Mangalitza | JN601069 | Domestic | Cannon et al. 2015. Anim Biotechnol. 26,17-28 |
| Mangalitza | JN601068 | Domestic | Cannon et al. 2015. Anim Biotechnol. 26,17-28 |
| Landrace | AY237526 | Domestic | Alves et al. 2003. Anim. Genet. 34,319-324. |
| Landrace | AY237527 | Domestic | Alves et al. 2003. Anim. Genet. 34,319-324. |
| Landrace | GU211931 | Domestic | Georgescu et al. 2012. Int J Mol Sci. 13,8467–8481 |
| Landrace | EU586546 | Domestic | Souza et al. 2009. Anim. Genet.40,759-62 |
| Landrace | EU586542 | Domestic | Souza et al. 2009. Anim. Genet.40,759-62 |
| Landrace | EU586539 | Domestic | Souza et al. 2009. Anim. Genet.40,759-62 |
| Landrace | EU586538 | Domestic | Souza et al. 2009. Anim. Genet.40,759-62 |
| Landrace | EU586516 | Domestic | Souza et al. 2009. Anim. Genet.40,759-62 |
| Large White | GU135804 | Domestic | Unpublished |
| Large White | GU135803 | Domestic | Unpublished |
| Large White | AB015079 | Domestic | Watanabe et al.1999. Mol. Ecol. 8,1509-1512. |
| Large White | GU211925 | Domestic | Georgescu et al. 2012. Int J Mol Sci. 13,8467–8481 |
| Large White | AY830188 | Domestic | Unpublished |
| Large White | EU586544 | Domestic | Souza et al. 2009. Anim. Genet.40,759-62 |
| Large White | EU586540 | Domestic | Souza et al. 2009. Anim. Genet.40,759-62 |
| Large White | AY237525 | Domestic | Alves et al. 2003. Anim. Genet. 34,319-324. |
| Large White | AY237524 | Domestic | Alves et al. 2003. Anim. Genet. 34,319-324. |
| Papua New Guinea | AM492646 | Feral | Mona. et al . 2007. Mol. Phylogenet. Evol. 45,757 |
| Papua New Guinea | AM492647 | Feral | Mona. et al . 2007. Mol. Phylogenet. Evol. 45,757 |
| Papua New Guinea | AM492648 | Feral | Mona. et al . 2007. Mol. Phylogenet. Evol. 45,757 |
| Papua New Guinea | AM492649 | Feral | Mona. et al . 2007. Mol. Phylogenet. Evol. 45,757 |
| Papua New Guinea | AM492650 | Feral | Mona S. et al .2007. Mol. Phylogenet. Evol. 45,757-762 |
| Portugal | AM492654 | Wild boar | Mona S. et al .2007. Mol. Phylogenet. Evol. 45,757-762 |
| Portugal | AM492653 | Wild boar | Mona S. et al .2007. Mol. Phylogenet. Evol. 45,757-762 |
| Russian | HM010461 | Wild boar | Ramayo et al. 2011. Anim. Genet. 42, 96-99. |
| Russian | HM010462 | Wild boar | Ramayo et al. 2011. Anim. Genet. 42, 96-99. |
| Russian | HM010463 | Wild boar | Ramayo et al. 2011. Anim. Genet. 42, 96-99. |
| Russian | HM010464 | Wild boar | Ramayo et al. 2011. Anim. Genet. 42, 96-99. |
| Russian | HM010465 | Wild boar | Ramayo et al. 2011. Anim. Genet. 42, 96-99. |
| Russian | HM010466 | Wild boar | Ramayo et al. 2011. Anim. Genet. 42, 96-99. |
| Russian | HM010467 | Wild boar | Ramayo et al. 2011. Anim. Genet. 42, 96-99. |
| Russian | HM010468 | Wild boar | Ramayo et al. 2011. Anim. Genet. 42, 96-99. |
| Russian | HM010469 | Wild boar | Ramayo et al. 2011. Anim. Genet. 42, 96-99. |
| Russian | HM010470 | Wild boar | Ramayo et al. 2011. Anim. Genet. 42, 96-99. |
| Russian | HM010471 | Wild boar | Ramayo et al. 2011. Anim. Genet. 42, 96-99. |
| Russian | HM010472 | Wild boar | Ramayo et al. 2011. Anim. Genet. 42, 96-99. |
| Russian | HM010473 | Wild boar | Ramayo et al. 2011. Anim. Genet. 42, 96-99. |
| Russian | HM010474 | Wild boar | Ramayo et al. 2011. Anim. Genet. 42, 96-99. |
| Russian | AY237496 | Wild boar | Ramírez et al. 2009. Mol Biol Evol. 26: 2061-72 |
| Russian | AY237496 | Wild boar | Ramírez et al. 2009. Mol Biol Evol. 26: 2061-72 |
| Russian | AY237496 | Wild boar | Ramírez et al. 2009. Mol Biol Evol. 26: 2061-72 |
| Spanish | EF061501 | Wild boar | Ramírez et al. 2009. Mol Biol Evol. 26: 2061-72 |
| Spanish | EF061500 | Wild boar | Ojeda et al. 2006. Genetics 174:2119-2127 |
| Spanish | EF061499 | Wild boar | Ojeda et al. 2006. Genetics 174:2119-2127 |
| Spanish | EF061498 | Wild boar | Ojeda et al. 2006. Genetics 174:2119-2127 |
| Spanish | EU531830 | Wild boar | Ojeda et al. 2006. Genetics 174:2119-2127 |
| Spanish | [AY237490](http://www.ebi.ac.uk/cgi-bin/sva/sva.pl?query=AY237490&search=Go&snapshot=) | Wild boar | Alves et al. 2003. Anim. Genet. 34, 319-324. |
| Spanish | [AY237491](http://www.ebi.ac.uk/cgi-bin/sva/sva.pl?query=AY237491&search=Go&snapshot=) | Wild boar | Alves et al. 2003. Anim. Genet. 34, 319-324. |
| Spanish | [AY237492](http://www.ebi.ac.uk/cgi-bin/sva/sva.pl?query=AY237492&search=Go&snapshot=) | Wild boar | Alves et al. 2003. Anim. Genet. 34, 319-324. |
| Spanish | [AY237493](http://www.ebi.ac.uk/cgi-bin/sva/sva.pl?query=AY237493&search=Go&snapshot=) | Wild boar | Alves et al. 2003. Anim. Genet. 34, 319-324. |
| Spanish | [AY237494](http://www.ebi.ac.uk/cgi-bin/sva/sva.pl?query=AY237494&search=Go&snapshot=) | Wild boar | Alves et al. 2003. Anim. Genet. 34, 319-324. |
| Spanish | [AY237495](http://www.ebi.ac.uk/cgi-bin/sva/sva.pl?query=AY237495&search=Go&snapshot=) | Wild boar | Alves et al. 2003. Anim. Genet. 34, 319-324. |
| Spanish | [AY237496](http://www.ebi.ac.uk/cgi-bin/sva/sva.pl?query=AY237496&search=Go&snapshot=) | Wild boar | Alves et al. 2003. Anim. Genet. 34, 319-324. |
| Spanish | [AY237497](http://www.ebi.ac.uk/cgi-bin/sva/sva.pl?query=AY237497&search=Go&snapshot=) | Wild boar | Alves et al. 2003. Anim. Genet. 34, 319-324. |
| Spanish | [AY237498](http://www.ebi.ac.uk/cgi-bin/sva/sva.pl?query=AY237498&search=Go&snapshot=) | Wild boar | Alves et al. 2003. Anim. Genet. 34, 319-324. |
| Spanish | [AY237499](http://www.ebi.ac.uk/cgi-bin/sva/sva.pl?query=AY237499&search=Go&snapshot=) | Wild boar | Alves et al. 2003. Anim. Genet. 34, 319-324. |

Supplementary Figure S1. Admixture analysis (K = 2-9) of Romanian wild boars and a previously published dataset (Manunza, A. et al. 2013. PLoS One 8,e55891) composed by populations from the Near East (Turkey, Iran and Armenia), East Europe (Russia), West Europe (Belgium and Spain) and Mangalitza and Iberian pigs. The cross-validation error technique showed that the most significant number of clusters was 4.


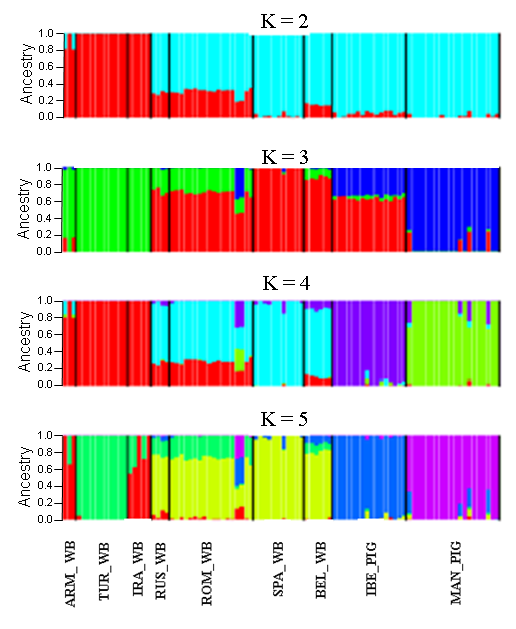


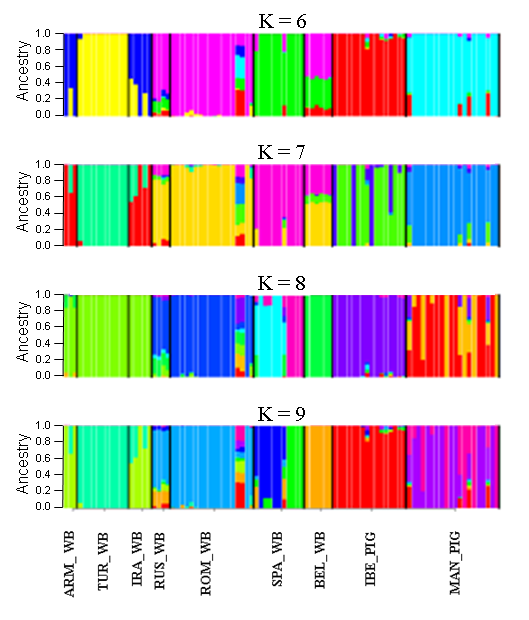

Supplement: Supplementary Information [file srep29913-s1.doc]
